# Is It Coincidence or Consequence for a Case with Antiphospholipid Antibody Syndrome Overlapping SLE to Develop an Immune Complex Nephropathy Followed by a Nonimmune Complex Podocytopathy?

**DOI:** 10.1155/2018/6746473

**Published:** 2018-07-24

**Authors:** Jinil Yoo, Hugo Villanueva, Manimaran Kaliamurthy, John Kang, Lin Lwin

**Affiliations:** ^1^Montefiore Medical Center, Wakefield Campus, Bronx, NY, USA; ^2^New York Medical College, Metropolitan Hospital, New York, NY, USA

## Abstract

Antiphospholipid antibody syndrome (APS) may occur in a primary form or in association with SLE and seldom presents with nephrotic syndrome (NS). We present a case with APS who developed recurrent NS 6 years apart. The first episode of NS occurred with biopsy findings consistent with lupus nephritis (LN) class V (membranous) with no clear evidence of SLE, and responded to a remission with steroids and MMF. On the 2^nd^ episode, the biopsy revealed negative immunofluorescent (IF) study for immune complexes and EM findings of complete effacement of foot processes and acellular debris in thickened capillary walls, compatible with healed previous episode of membranous LN and minimal change disease (MCD), a nonimmune complex podocytopathy. The 2^nd^ episode responded to a partial remission, primarily with a short-term steroid therapy, and subsequently developed serologic evidence of SLE. Now there is growing evidence that a subset of SLE patients with NS are found to have MCD, likely due to podocyte injury caused by nonimmune complex pathway, called lupus podocytopathy. In LN, serial kidney biopsies often show transformation from one to another class of immune complex-induced glomerular lesions; however there are rare reports describing transformation of an immune complex to a nonimmune complex LN. Since the pathogenic mechanism of lupus podocytopathy is not delineated, and so far there are no reports on transformation of membranous LN, an immune complex nephropathy, to a nonimmune complex lupus podocytopathy, it still remains as a question whether our case with APS overlapping SLE had a concomitant membranous LN and lupus podocytopathy, or consequential membranous LN and lupus podocytopathy 6 years apart.

## 1. Introduction

We present a case with antiphospholipid antibody syndrome (APS) who developed recurrent nephrotic syndrome (NS) 6 years apart. The first episode occurred with biopsy findings consistent with lupus nephritis (LN) class V (membranous) without clinical evidences of systemic lupus erythematosus (SLE), and the second episode occurred with pathologic findings of a nonimmune complex podocytopathy on the repeat kidney biopsy, subsequently with positive serologic evidences of SLE.

## 2. Case Presentation

**Figure 1:**
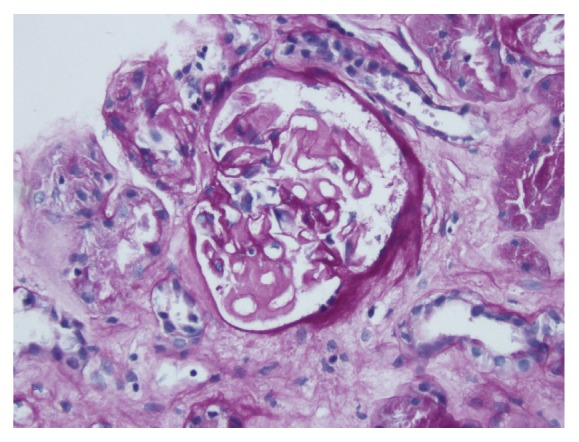
Thickened mesangium and capillary loops PAS 200x.

**Figure 2:**
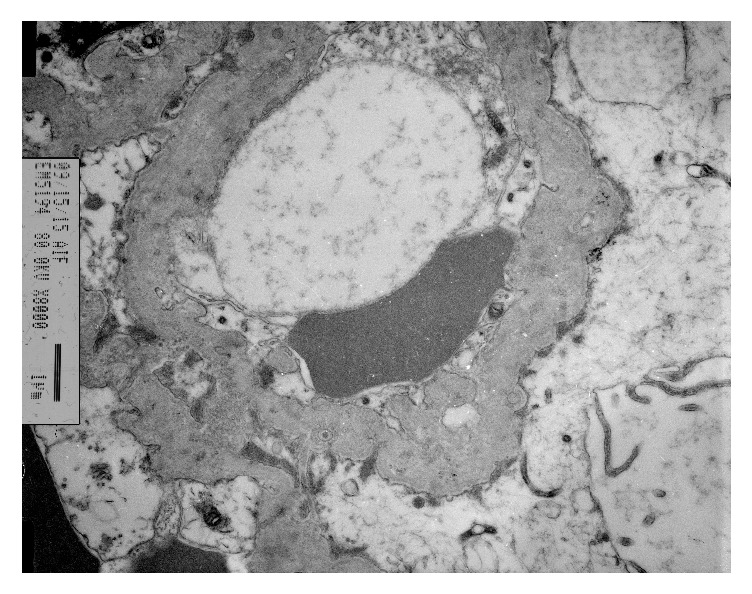
Thickened capillary walls with acellular debris, total foot process effacement on EM.

An African American man with the diagnosis of APS at age 18 was admitted with thrombosis of inferior vena cava and left renal vein, and found to have NS (16 g of protein in 24 h urine and serum albumin 1.7 g/dL) at age 25. The kidney biopsy revealed findings consistent with lupus nephritis (LN) class V (membranous). He had a brief episode of thrombocytopenia and prolonged PTT and negative serology for SLE except one positive ANA. He was treated with prednisone (up to 40 mg p.o. daily), mycophenolate mofetil (MMF) up to 1.5 g p.o. bid, enalapril, simvastatin, and warfarin. The follow-up visit in 4 months revealed serum creatinine (S_cr_) 1.2 mg/dL (0.11 mmol/L), serum albumin (S_alb_) 3.4 g/dL, and 0.6 g protein in 24 h urine. Six years later, at age 31, he presented with recurrent NS (11.5 g of protein in 24 h urine and S_alb_ 2.2 g/dL), and without any recent allergic reaction or exposure to NSAIDs. Physical exam revealed BP 130/85 mmHg, weight 121.8 kg, height 198 cm, no abnormal skin lesions, but 2+ pitting edema of both lower legs; otherwise there were no remarkable findings. Lab studies showed Hgb of 12.3 g/dL, Hct 36.4%, WBC 6,600 /*μ*L and platelet 154,000 /*μ*L, urine microscopic exam with RBC 21-50/HPF, BUN 24 mg/dL (4 mmol/L), S_cr_ 1.1 mg/dL (0.1 mmol/L), negative SLE serology (ANA, anti-dsDNA, anti-Smith, cardiolipin antibody), C_3_ 84 (N:85-288), C_4_ 24 (N:17-64), CH_50_ 45 (N: 31-60), and positive beta-2 glycoprotein 1 (IgA). Under the impression of recurrent membranous LN, he was placed on methylprednisolone (48 mg p.o. daily) and MMF (1.5 g p.o. bid) and enalapril (10 mg p.o. daily) and then developed disseminated herpes zoster (HZ) in 4 weeks. MMF was stopped and he was started on IV acyclovir and continued on methylprednisolone (Medrol) 24 mg p.o. daily. Upon resolution of HZ, a second biopsy was performed with findings of thickened capillary walls with lucencies, moderate mesangial matrix expansion, and no podocytes abnormality on the light microscopy (LM) (*Figure 1)*, and negative immunofluorescent study (IF) for IgG, IgA, C3, C1q, light chains, and faint IgM on IF, and electronmicroscopy (EM) with complete (90-100%) effacement of foot processes, acellular debris in thickened capillary walls, and sparse immune deposits in mesangium* (Figure 2)*, which were consistent with healed membranous nephropathy (MN) and minimal change disease- (MCD-) like process. After his discharge from the hospital on Medrol 24 mg daily, enalapril, warfarin, and intermittent use of furosemide, he did not come for follow-up, stating “no more swelling of legs” and “I am feeling fine.” Four months after discharge (not on Medrol for 3 months), he came for lab tests, which showed S_cr_ 1.37 mg/dL (1.21 mmol/L), S_alb_ 3.0 g/dL, 3.2 g of protein in 24 h urine, positive ANA (1:80), and positive anti-ds DNA.

## 3. Discussion

It is known that APS, an autoimmune disease, may occur as a primary form without any underlying disease or in association with SLE [1], and that 37% of patients with SLE have positive beta-2-glycoprotein 1 antibodies [2]. In the primary APS, features of thrombotic microangiopathy (TMA) are the most characteristic lesions of APS nephropathy [3]; nephrotic syndrome (NS) is rare, but described with presence of thrombosis of renal vein [4].

In our case with the diagnosis of APS at age 18, the first episode of NS at age 25 was found with biopsy findings consistent of membranous lupus nephropathy (LN Class V) and his NS responded to remission with steroid and MMF therapy. At age 31 (six years later), the second episode of NS occurred, and the second kidney biopsy revealed negative IF for IgG, IgA, C3, and C1q, except faint IgM, with EM findings of complete effacement of foot processes and acellular debris in thickened capillary walls compatible with the diagnosis of a nonimmune complex podocytopathy and healed previous episode of membranous LN. The second episode of NS with minimal change disease/lupus podocytopathy responded to partial remission, primarily to a short-term steroid therapy.

A subset of SLE patients with NS have been found to have no evidence of immune complex deposition or endocapillary proliferation/inflammation on kidney biopsy, comparable to minimal change disease (MCD), and due to podocyte injury caused by nonimmune complex pathway called lupus podocytopathy [5, 6].

Rare case reports of MCD in SLE started to appear in Japanese literature (1984) and English literature (1995) [5]. In 2002, Dube et al. [5] reported the clinical and pathologic findings in 7 patients with SLE and MCD, and highlighted the entity as an underrecognized and highly reversible form of NS in patients with SLE. In 2005, Kraft et al. [6] reported the development of nephrotic range proteinuria in 8 SLE patients with no evidence of peripheral capillary immune complex deposition and called it glomerular podocytopathy. In 2016, Hu et al. [7] reported 50 cases of lupus podocytopathy (minimal change in 13 cases, mesangial proliferation in 28 cases, and FSGS in 9 cases) from 3,750 biopsies of SLE patients, and described different responses to treatment and its outcomes, in parallel with the underlying histologic patterns.

The pathogenic mechanism of MCD/lupus podocytopathy is not well delineated, even though abnormal T-cell mediated immune responses have been implicated in idiopathic MCD [8].

Now there is growing evidence for glomerular podocytes as immunologically active cells. Podocytes can have upregulated B7-1 molecule involved in T-cell costimulation by lipopolysaccharide (LPS) via TLR4 and lead to proteinuria by altering slit diaphragm [9], and podocytes also act as antigen presenting cells, participating in immune-mediated glomerular diseases [10].

In our case, the first episode of nephrotic syndrome occurred with membranous LN (Class V), an immune complex nephropathy; then a second episode occurred six years later with lupus podocytopathy, a nonimmune complex nephropathy. In LN, serial kidney biopsies often show transformation from one to another class of immune complex-induced glomerular lesions. There is also one case report [11] describing a 45-year-old lady with LN Class III (focal proliferative) who developed NS 5 years later, and had histologic changes consistent with MCD on the repeat biopsy. It could mean that lupus podocytopathy may occur in a healed late stage of an immune complex-induced lupus nephritis. However, so far no reports of transforming membranous LN, immune complex nephropathy, to a nonimmune complex lupus podocytopathy are found in English literature. It is reported that relapse or worsening of NS in idiopathic membranous nephropathy can occur even though the glomerular immune deposits have been eradicated [12], and nephrotic range proteinuria in patients with membranous LN was better correlated with the degree of foot process effacement than the degree of immune complex deposition, pointing to it as manifestation of concomitant podocyte dysfunction [13].

## 4. Conclusion

Since the pathogenic mechanism of lupus podocytopathy is not delineated, and so far there are no reports on transformation of membranous LN, an immune complex nephropathy, to a nonimmune complex lupus podocytopathy, it still remains as a question whether our case with APS overlapping SLE had a concomitant membranous LN and lupus podocytopathy, or consequential membranous LN and lupus podocytopathy 6 years apart.
